# Supplementary material for: Multi‐dataset identification of innovative feature genes and molecular mechanisms in keratoconus
Source: J Cell Mol Med. 2024 Sep 19;28(18):e70079. doi: 10.1111/jcmm.70079 (PMC11412914; doi:10.1111/jcmm.70079)
Supplement: Supplementary file 2 — Material S2. [file JCMM-28-e70079-s002.zip › 7.GSEA analysis/4.MYRF/KEGG_PENTOSE_AND_GLUCURONATE_INTERCONVERSIONS.html]

Details for gene set KEGG\_PENTOSE\_AND\_GLUCURONATE\_INTERCONVERSIONS[GSEA]

|  || Dataset | MYRF.group.cls#h\_versus\_l |
| Phenotype | group.cls#h\_versus\_l |
| Upregulated in class | h |
| GeneSet | KEGG\_PENTOSE\_AND\_GLUCURONATE\_INTERCONVERSIONS |
| Enrichment Score (ES) | 0.29956603 |
| Normalized Enrichment Score (NES) | 0.8092586 |
| Nominal p-value | 0.74949086 |
| FDR q-value | 1.0 |
| FWER p-Value | 1.0 |
Table: GSEA Results Summary

  

Fig 1: Enrichment plot: KEGG\_PENTOSE\_AND\_GLUCURONATE\_INTERCONVERSIONS      
 Profile of the Running ES Score & Positions of GeneSet Members on the Rank Ordered List

  

| SYMBOL | TITLE | RANK IN GENE LIST | RANK METRIC SCORE | RUNNING ES | CORE ENRICHMENT || 1 | UGT1A8 | na | 369 | 0.620 | 0.2008 | Yes |
| 2 | UGT1A6 | na | 2556 | 0.320 | 0.2470 | Yes |
| 3 | UGT2B4 | na | 3822 | 0.260 | 0.2993 | Yes |
| 4 | UGT2B11 | na | 6521 | 0.178 | 0.2821 | Yes |
| 5 | UGT2B28 | na | 7657 | 0.147 | 0.2996 | Yes |
| 6 | RPEL1 | na | 9091 | 0.114 | 0.2972 | No |
| 7 | DCXR | na | 10851 | 0.081 | 0.2740 | No |
| 8 | UGT2B10 | na | 12166 | 0.061 | 0.2570 | No |
| 9 | UGT2A3 | na | 16075 | 0.017 | 0.1502 | No |
| 10 | UGT2B7 | na | 16473 | 0.013 | 0.1433 | No |
| 11 | RPE | na | 17765 | 0.002 | 0.1066 | No |
| 12 | GUSB | na | 18367 | -0.004 | 0.0907 | No |
| 13 | CRYL1 | na | 18930 | -0.010 | 0.0778 | No |
| 14 | UGP2 | na | 19571 | -0.017 | 0.0651 | No |
| 15 | UGDH | na | 19767 | -0.019 | 0.0660 | No |
| 16 | UGT2B15 | na | 26025 | -0.113 | -0.0757 | No |
| 17 | UGT2A1 | na | 29490 | -0.205 | -0.1054 | No |
| 18 | XYLB | na | 29670 | -0.212 | -0.0382 | No |
| 19 | DHDH | na | 29820 | -0.218 | 0.0318 | No |
| 20 | AKR1B1 | na | 32075 | -0.321 | 0.0764 | No |
Table: GSEA details [plain text format]

  

Fig 2: KEGG\_PENTOSE\_AND\_GLUCURONATE\_INTERCONVERSIONS      
 Blue-Pink O' Gram in the Space of the Analyzed GeneSet

  

Fig 3: KEGG\_PENTOSE\_AND\_GLUCURONATE\_INTERCONVERSIONS: Random ES distribution      
 Gene set null distribution of ES for **KEGG\_PENTOSE\_AND\_GLUCURONATE\_INTERCONVERSIONS**
